# *SDG712*, a Putative H3K9-Specific Methyltransferase Encoding Gene, Delays Flowering through Repressing the Expression of Florigen Genes in Rice

**DOI:** 10.1186/s12284-021-00513-9

**Published:** 2021-08-06

**Authors:** Siju Zhang, Hongjiao Hao, Xiaonan Liu, Yingying Li, Xuan Ma, Weiyin Liu, Rui Zheng, Shanshan Liang, Weijiang Luan

**Affiliations:** College of Life Sciences, Tianjin Key Laboratory of Animal and Plant Resistance, Tianjin Normal University, Tianjin, 300387 China

**Keywords:** Rice, *SDG712*, Flowering, Histone methyltransferase, Florigen

## Abstract

SET domain group (SDG) proteins have been identified to be involved in histone modification and participate in diverse biological processes. Rice contains 41 *SDG* genes, however, most of which have not been functionally characterized. Here, we report the identification and functional investigation of rice *SDG712* gene. Phylogenic analysis revealed that SDG712 belongs to the H3K9-specific SDG subclade. *SDG712* is highly expressed in leaves during reproductive growth stage with obvious circadian rhythmic pattern. Mutation of *SDG712* promotes rice flowering, while overexpression of *SDG712* delays rice flowering. Gene expression analysis suggested that *SDG712* acts downstream of *Hd1*, while acts upstream of *Ehd1*, *Hd3a and RFT1*. Subcellular localization assay demonstrated that SDG712 is localized in the nucleus. Chromatin immunoprecipitation (ChIP) assay showed that the H3K9me2 levels at *Hd3a* and *RFT1* loci were increased in *SDG712* overexpression transgenic plants, indicating that SDG712 may mediate the H3K9 di-methylation on these loci to repress rice flowering. Taken together, our findings demonstrated that *SDG712* is a negative flowering regulatory gene in rice, and it delays flowering through repressing key flowering regulator gene *Ehd1* and the florigen genes *Hd3a and RFT1*.

**Supplementary Information:**

The online version contains supplementary material available at 10.1186/s12284-021-00513-9.

## Background

Rice is an important food crop worldwide, which supplies more than half of the world’s population. Rice yield is restricted by various environmental factors, including photoperiod, temperature, nutrition, etc. Heading-date (or flowering time) is an important agronomic trait closely related to rice yield and regional adaptability; it is mainly modulated by photoperiodic signals and endogenous flowering regulatory genes. Rice is a facultative short-day (SD) plant, short-day induces the flowering while long-day (LD) represses the flowering. In the past decades, with the development of molecular genetics, several key genes have been identified to play important roles in photoperiodic flowering regulatory pathways in rice. *Hd3a* and *RFT1* are two important promoting genes of rice flowering (Komiya et al., 2008). They encode the florigens, a kind of small globular protein, synthesized in leaves, and transported into shoot apical meristem (SAM) cells, to promote rice flowering under SD and LD conditions, respectively. In SAM cells, florigens bind with 14–3-3 and OsFD1 protein, forming the florigen activation complex (FAC). Then, FAC binds to the promoter of floral identity genes, such as *MADS14* and *MADS15*, to induce the floral initiation (Taoka et al., 2011, 2013).

The expression of florigen genes are mainly regulated by several critical genes under SD and LD conditions. Under SD condition, *Hd1* encodes a zinc finger protein and acts as the upstream regulator to positively regulate the expression of florigen gene *Hd3a* (Yano et al., 2000). *Ehd1* encodes a B-type response regulator and up-regulates the expression of *Hd3a* to promote rice flowering under SD condition (Doi et al., 2004). Under LD condition, on the one hand, *Hd3a* was repressed by *Hd1* which serves dual functions with promoting rice flowering under SD and inhibiting rice flowering under LD conditions. On the other hand, *Ghd7* acts as a key repressor of *Hd3a* to inhibit rice flowering under LD condition (Xue et al., 2008; Zheng et al., 2019). Recent studies showed that *DTH8* encodes the HAP3 subunit of HF-YB protein and interacts with Hd1 and Ghd7 to repress the expression of *Hd3a* by the formation of Hd1-DTH8-Ghd7 complex (Cai et al., 2019; Wei et al., 2010). When the expression of *Hd3a* is repressed, *RFT1* serves as the major florigen gene to promote rice flowering under LD condition. *RFT1* is induced by *Ehd1* and *DTH2* under LD condition (Komiya et al., 2008; Wu et al., 2013). *Ehd1* was negatively regulated by *Hd1*, *Ghd7*, *DTH8 and DTH7*, and positively regulated by *Ehd2*, *Ehd4* and *Ehd3* (Xue et al., 2008; Wei et al., 2010; Gao et al., 2014; Matsubara et al., 2008, 2011; Gao et al., 2013).

In recent years, epigenetic regulation was reported to play important roles in plant growth and development (Jacob et al., 2009; Guo et al., 2010; Sui et al., 2012). In eukaryotic cells, lysine residues (K) of histone H3 subunit were prone to be acetylated or methylated. Acetylation of lysine residues generally promotes gene transcription, while methylation of different lysine residues has diversified effects (Thorstensen et al., 2011). In general, methylation of H3K4 and H3K36 promotes gene expression, while methylation of H3K9 and H3K27 represses gene expression (Thorstensen et al., 2011). The methylation process of lysine residues on H3 is conducted by histone methyltransferases (HMTase), and the methylation activity of HMTase is site-specific. The *SET domain group* (*SDG*) genes were identified to perform the activity of HMTase (Ng et al., 2007). Several *SDGs* were reported to be involved in the photoperiodic flowering pathways. In *Arabidopsis*, *AtSDG25* and *AtSDG26* both encode H3K4/H3K36-specific HMTases. *AtSDG25* represses flowering by activating the expression of *FLOWERING LOCUS C,* and *AtSDG26* promotes flowering by binding at *SOC1* locus (Berr et al., 2009, 2015). *AtSDG27* encodes a H3K4-specific HMTase and is involved in mediating the H3K4 tri-methylation at *FLOWERING LOCUS C* locus (Pien et al., 2008). *AtSDG8* encodes a H3K36-specific HMTase involving in H3K36 di-methylation at *FLOWERING LOCUS C* (Zhao et al., 2005). In rice, *SDG701* and *SDG723* encode H3K4-specific HMTases, promoting flowering by enhancing the expression of florigen genes and *Ehd3* (Choi et al., 2014, Liu et al., 2017). SDG723 also mediates the H3K4 tri-methylation at *Ehd1* locus by interacting with OsWD5a and SIP1 (Jiang et al., 2018a, b). *SDG708*, *SDG724* and *SDG725* encode H3K36-specific HMTases and promote rice flowering by inducing the expression of *Ehd1*, *Hd3a* and *RFT1* gene under SD and LD conditions (Sun et al., 2012; Sui et al., 2013; Liu et al., 2016). *SDG711* and *SDG718* both encode H3K27-specific HMTases to repress the expression of *OsLF* gene (a repressor of *Hd1*). *SDG711* delays flowering under LD condition, and *SDG718* promotes flowering under SD condition (Liu et al., 2014).

Rice genome contains 41 *SDGs*, most of which have not been functionally characterized, and no H3K9-specific *SDGs* have been reported to be involved in flowering regulation in rice (Shi et al., 2015). Here, we report that *SDG712* is a putative H3K9-specific HMTase encoding gene and is a negative regulator of rice flowering. Loss of function of *SDG712* promotes flowering while overexpression of *SDG712* inhibits rice flowering. The key flowering regulatory gene *Ehd1* and florigen genes *Hd3a* and *RFT1* are down-regulated in *SDG712* overexpression transgenic lines, suggesting that *SDG712* delays rice flowering through repressing key flowering regulator *Ehd1* and the florigen genes *Hd3a and RFT1*.

## Results

### SDG712 Is a Putative H3K9-Specific methyltransferase

**Fig. 1:**
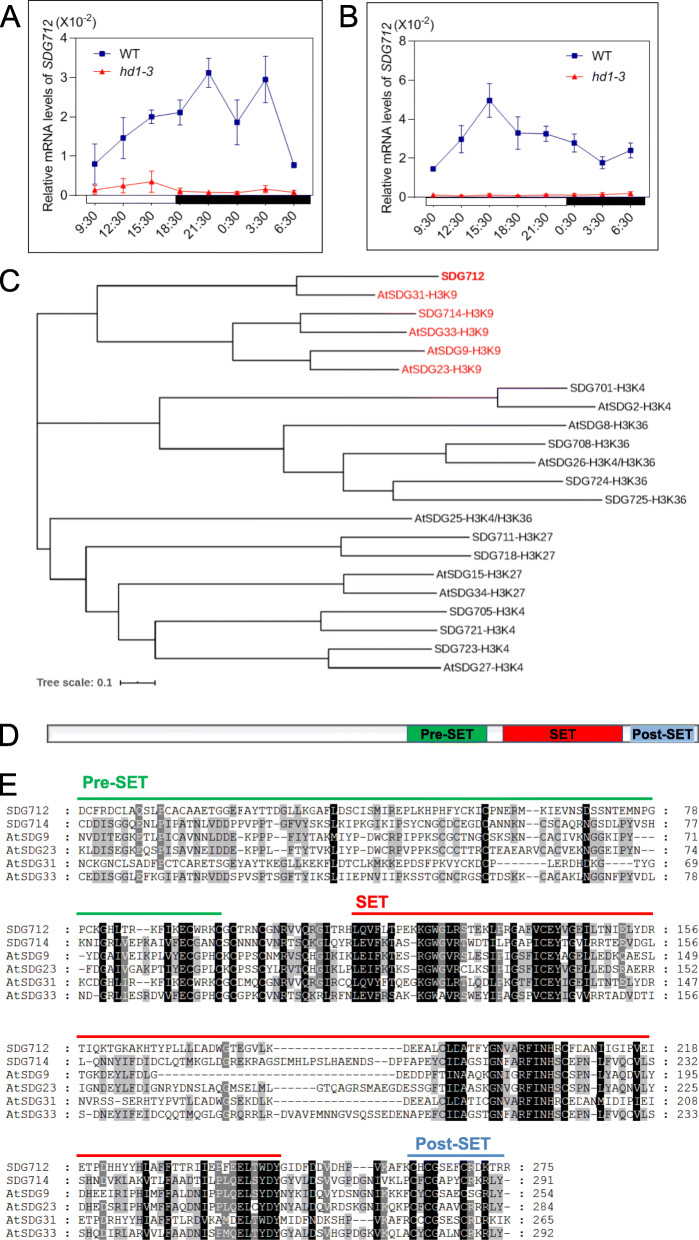
Identification and phylogenic analysis of SDG712. **A** and **B**, Relative expression levels of *SDG712* in Zhonghua11 and *hd1–3* under short-day (SD) and long-day (LD) conditions, respectively. WT, Zhonghua11. White boxes indicate light-period, and filled boxes indicate dark-period. **C**, Phylogenic analysis of SDG712 and functionally characterized SDGs in *Arabidopsis* and rice using MEGA-X. The Genebank accession numbers for the peptide sequences of SDGs are NP_193253.4 (AtSDG2), NP_177854.6 (AtSDG8), NP_001324386.1 (AtSDG9), NP_001078559.1 (AtSDG15), NP_850030.1 (AtSDG23), NP_001318731.1 (AtSDG25), NP_177797.2 (AtSDG26), NP_850170.1 (AtSDG27), NP_187088.2 (AtSDG31), NP_196900.1 (AtSDG33), NP_197821.1 (AtSDG34), XP_015649923.1 (SDG701), XP_015621708.1 (SDG705), XP_015633505.1 (SDG708), XP_015644234.1 (SDG711), XP_015623394.1 (SDG712), XP_015629359.1 (SDG714), XP_015630972.1 (SDG718), XP_015611850.1 (SDG721), XP_015612383.1 (SDG723), XP_015651319.1 (SDG724), and XP_015625429.1 (SDG725). **D**, The common motifs of characterized H3K9-specific SDGs and SDG712. Pre-SET, SET and Post-SET denote three conserved domains in SDG families. **E**, Amino acids sequence alignment of SDG712 and characterized H3K9-specific SDGs in conserved Pre-SET, SET and Post-SET domains

In our previous studies, we identified a photoperiod-insensitive mutant *hd1–3*, in which *Hd1* gene was functionally deficient due to several deletions/insertions (Luan et al., 2009). To identify novel genes involved in flowering regulation, we performed microarray analysis to screen for the up- and down-regulated genes affected by *Hd1* mutation. The majority of genes do not have significantly differential expressions between the wild-type and the *hd1–3* mutant under either SD or LD condition (red bracket in Supplemental Fig. 1A). We identified 264 genes that showed above 2-fold expression changes in both SD and LD conditions (Supplemental Table 1). Gene ontology analysis of these 264 genes showed that they were enriched in protein kinase, protein modification and degradation, and phosphorylation processes (Supplemental Fig. 1B). Among them, 44 genes have above 5-fold changes in both LD and SD conditions (Supplemental Fig. 1C). We focused on the gene *LOC_Os02g40770* (red asterisk in Supplemental Fig. 1C), which encodes a putative methyltransferase and was designated as *SDG712* in a previous phylogenetic study (Ng et al., 2007). qRT-PCR assay confirmed that the expression of *SDG712* was significantly decreased in *hd1–3* mutant compared with wild type under both SD and LD conditions (Fig. 1A, B), in agreement with the microarray result.

BLAST analysis showed that SDG712 belongs to the histone methyltransferase protein family containing SET domain. Phylogenic analysis of SDG712 and several functionally characterized SDGs in rice and *Arabidopsis* showed that SDG712 was grouped into H3K9-specific subclade (Fig. 1C). This subclade also includes *Arabidopsis* AtSDG9, AtSDG23, AtSDG31, AtSDG33 and rice SDG714 (Veiseth et al., 2011; Ebbs and Bender, 2006; Ding et al., 2007). The closest homolog of SDG712 in *Arabidopsis* is AtSDG31, which converts H3K9me1 to H3K9me3 on transposon chromatin in *Arabidopsis* (Veiseth et al., 2011). The H3K9-specific subclade members contain a common classic SET domain that is featured by Pre-SET, SET and Post-SET domains (Ng et al., 2007; Ding et al., 2007) (Fig. 1D). These SDGs shows high similarity among the three domain (Fig. 1E), indicating that they may have common catalytic activity.

### *SDG712* Displays High Expression in Leaves during the Reproductive Growth Stage

**Fig. 2:**
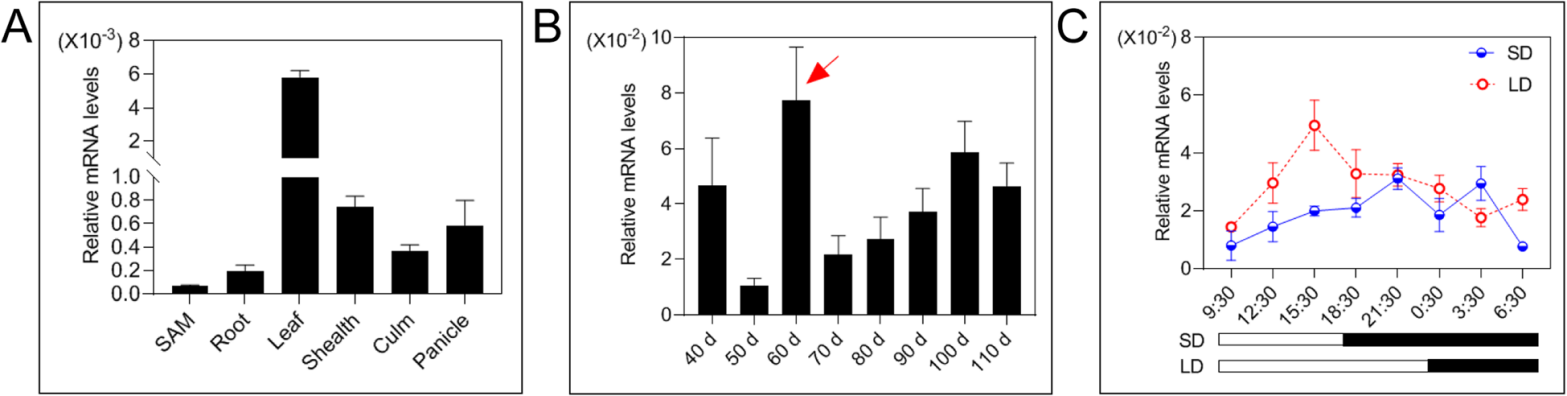
Spatial and temporal expression analysis of *SDG712* gene. **A**, The expression of *SDG712* in various tissues. **B**, The expression of *SDG712* at different growing stages. Arrow head indicates the transition stage from vegetative growth to reproductive growth. **C**, The diurnal expression pattern of *SDG712* under short-day and long-day conditions. SD, short-day condition; LD, long-day condition. White boxes indicate light-period, and filled boxes indicate dark-period

To investigate the temporal and spatial expression pattern of *SDG712*, wild type Zhonghua11 plants were grown in paddy field and various tissues were collected for qRT-PCR assay. The results showed that *SDG712* was expressed in all tissues examined, including roots, culms, sheaths, panicles and shoot apical meristems (SAMs), especially highly expressed in leaves (Fig. 2A). We further examined the expression levels of *SDG712* in leaves at different growth stages. The result showed that *SDG712* displays lower expression in the vegetative growth stage. *SDG712* exhibits higher expression during the transition phase from vegetative growth to reproductive growth (Fig. 2B), then maintains higher expression after flowering, especially during the ripening stages. Furthermore, we investigated the diurnal expression rhythm of *SDG712* in leaves in 24 h cycle under SD and LD conditions. The results showed that *SDG712* exhibits diurnal expression rhythm with the peak at daytime under LD condition and the peak at dark under SD condition (Fig. 2C), and the expression under LD condition is slightly higher than that under SD condition.

### Loss of Function of *SDG712* Promotes Heading-Date in Rice

**Fig. 3:**
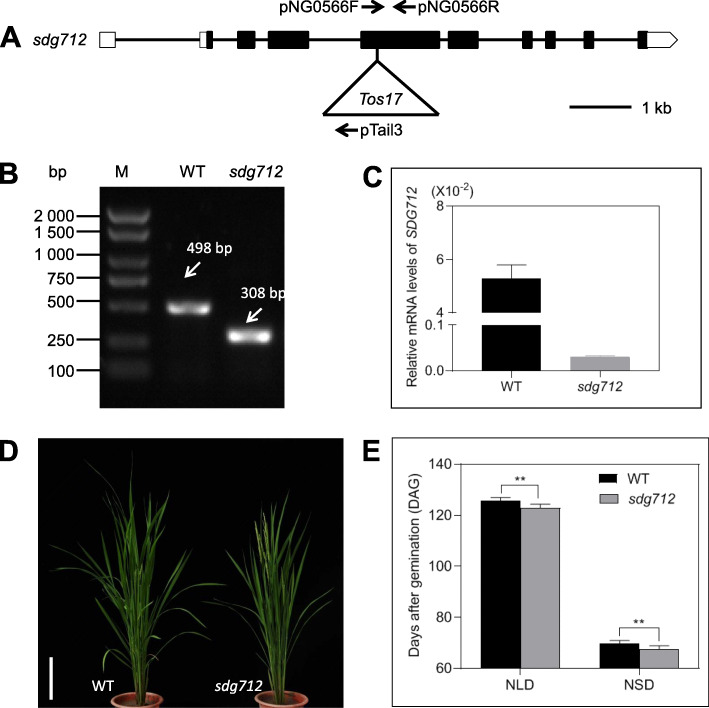
Identification and phenotypic analysis of *sdg712*. **A**, Schematic of *Tos17* insertion site in *sdg712* mutant. Open boxes denote 5’UTR and 3’UTR; filled boxes denote exons. Lines are introns, and arrows denote primers of detection. **B**, Identification of homozygous *sdg712* mutant using primers pNG0566F, pNG0566R and pTail3 (indicated by arrows in **A**). Primers pNG0566F and pNG0566R are located in the fourth exon of *sdg712* and pTail3 is located in the left border of *Tos17*. M, DNA ladder. WT is Nipponbare. **C**, The expression of *SDG712* in *sdg712* mutant and WT Nipponbare. *OsActin1* was used as internal control. **D**, The phenotype of *sdg712* under natural long-day condition. Bar = 20 cm. WT is Nipponbare. **E**, Heading-date of *sdg712* under natural long-day (NLD) and natural short-day (NSD) conditions. Values are shown as means ± SD. *N* ≥ 30 individual plants. WT is Nipponbare. Asterisks indicate statistically significant differences of t-test (**, *P* < 0.01)

To investigate the phenotype of loss of function of *SDG712*, we obtained a *SDG712 Tos17*-insertion line, *NG0566*, from the rice mutant library (http://tos.nias.affrc.go.jp) (Miyao et al., 2007), which was generated in the japonica variety Nipponbare. In *NG0566*, a ~ 4000 bp *Tos17* fragment was inserted into the fourth exon of *SDG712* (Fig. 3A)*,* resulting in the loss of function of *SDG712*. Therefore, we termed *NG0566* as *sdg712*. We further identified the homozygous *sdg712* mutant by tri-primer PCR and found that wild type plants can only amplify a 498 bp band by gene-specific primers, whereas homozygous *sdg712* mutants can only obtain a 308 bp band by the primer of *Tos17* border and gene-specific primer (Fig. 3A, B), suggesting that *Tos17* fragment indeed inserts into *SDG712*. We also examined the transcript level of *SDG712* in *sdg712* mutant by qRT-PCR. The results showed that the expression of *SDG712* was almost undetectable in the *sdg712* mutant (Fig. 3C), indicating that the *Tos17* insertion not only changed the reading-frame of *SDG712* but also reduced the transcript level of *SDG712*, thus *sdg712* is certainly a loss of function mutant.

To investigate the phenotype of *sdg712* mutant, *sdg712* homozygous mutant and the wild type Nipponbare were grown in Tianjin (117°E, 39°N; natural long-day condition, NLD) and Lingshui, Hainan province (110°E, 18°N; natural short-day condition, NSD) for heading-date observation. For plants grown in Tianjin, the *sdg712* plants flowered at 123 days after germination (DAG), while the wild type Nipponbare flowered at 126 DAG (Fig. 3D-E). For plants grown in Lingshui, the *sdg712* plants flowered at 68 DAG, while the wild type Nipponbare flowered at 70 DAG (Fig. 3E). These data showed that the loss of function of *SDG712* promotes rice heading, indicating that *SDG712* is a negative regulator of heading-date. In addition, agronomic traits including plant height, panicle length, grain numbers per panicle, and 1000-grain weight were also investigated in NLD fields of Tianjin, no significant difference between the wild type and the mutant was observed (Supplemental Fig. 2).

### Overexpression of *SDG712* Delays Heading-Date in Rice

**Fig. 4:**
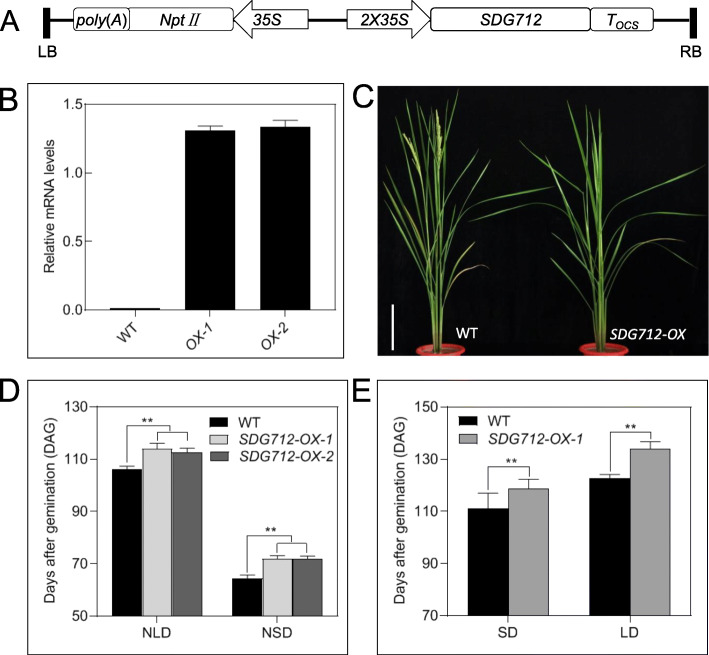
Obtainment and phenotypic analysis of *SDG712* overexpression transgenic lines. **A**, Structure of the vector used for overexpression of *SDG712*. 35S, 2X35S, *Cauliflower Mosaic Virus* (*CaMV*) 35S promoter sequences; *NptII*, neomycin phosphotransferase II gene*; T*_*OCS*_*,* terminator of octopine synthase gene; RB, right border; LB, left border. **B**, Expression of *SDG712* in transgenic plants overexpressing *SDG712* (*SDG712-OX*) by qRT-PCR. WT, Zhonghua11; *OX-1, OX-2*, transgenic lines. **C**, The phenotype of *SDG712-OX* under natural long-day condition*.* Bar = 20 cm. **D**, Heading-date of *SDG712-OX* lines under natural long-day (NLD) and natural short-day (NSD) conditions. Values are shown as means ± SD. N ≥ 30 individual plants. **E**, Heading-date of *SDG712-OX-1* plants under artificial day-length conditions. The wild type Zhonghua11 and *SDG712-OX1* transgenic plants were grown in Tianjin. Two-week-old plants were subjected to light-controlled treatments with 9 h light/ 15 dark for SD condition and 15 h light/9 h dark for LD condition. The heading-date was recorded after heading. Values are shown as means ± SD. *N* ≥ 20 individual plants. SD, artificial short-day condition; LD, artificial long-day condition. Asterisks in (**D**) and (**E**) indicate statistically significant differences of t-test (**, *P* < 0.01)

In order to investigate the biological function of *SDG712*, we constructed an overexpression vector with double *CaMV* 35S promoter to drive *SDG712* expression and obtained transgenic plants via *Agrobacterium*-mediated transformation method (Fig. 4A). Four independent transgenic lines were generated. Two independent transgenic lines were planted to produce homozygous T_2_ generation plants. qRT-PCR detection showed that *SDG712* expression levels of the two transgenic lines were significantly elevated compared with wild type plants (Fig. 4B), suggesting that overexpression vector worked well. Phenotypic observation found that the two transgenic lines both displayed a delayed heading-date phenotype in field (Fig. 4C-D). The heading-date of transgenic lines delayed about a week compared with the wild type Zhonghua11 under NLD condition in Tianjin (117°E, 39°N) and NSD condition in Lingshui (110°E, 18°N) (Fig. 4D). We also investigated several other agronomic traits of the wild type Zhonghua11 and *SDG712-overexpression* transgenic lines (*SDG712-OX* lines) in NLD fields of Tianjin, however, no significant difference was observed (Supplemental Fig. 2), demonstrating that *SDG712* may play roles only in heading-date regulation. Furthermore, we also investigated the heading-date of the wild type and the transgenic plants under artificial day-length conditions with 9 h light/ 15 h dark for SD condition and 15 h light/9 h dark for LD conditions. Under artificial SD condition, the heading-date of wild type was 111 DAG and that of *SDG712-OX1* was 119 DAG. Under artificial LD condition, the heading-date of wild type was 123 DAG and that of *SDG712-OX1* was 134 DAG (Fig. 4E). This result is in agreement with the result under NLD and NSD conditions, indicating that overexpression of *SDG712* represses rice flowering regardless of day-length conditions.

### *SDG712* Delays Rice Flowering through Inhibiting the Expression of Florigen Genes *Hd3a* and *RFT1*

**Fig. 5:**
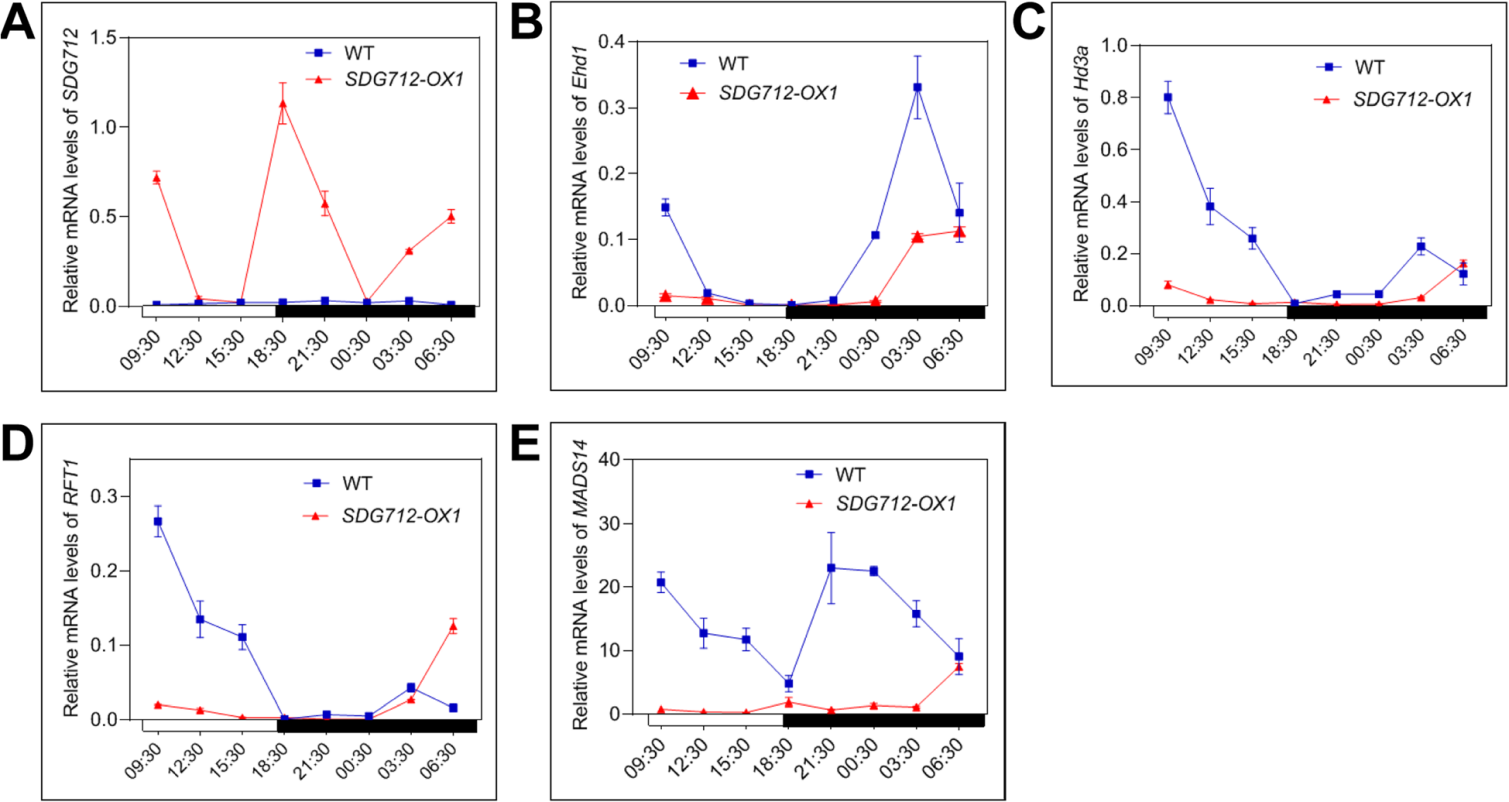
Gene expression under short-day (SD) condition. Plants were grown in artificial climate cabinets under SD condition with 9 h light/15 h dark at 28 °C. Penultimate leaves were collected for RNA extraction every 3 h within 24 h from 50-day-old plants. Os*Actin1* gene was used as internal control. WT, Zhonghua11. **A**, Transcript levels of *SDG712* in WT and *SDG712-OX1* plants. **B**-**E**, the expression of *Ehd1*, *Hd3a*, *RFT1* and *MADS14* in WT and *SDG712-OX1* plants. Open boxes denote light-period and filled boxes denote dark-period

**Fig. 6:**
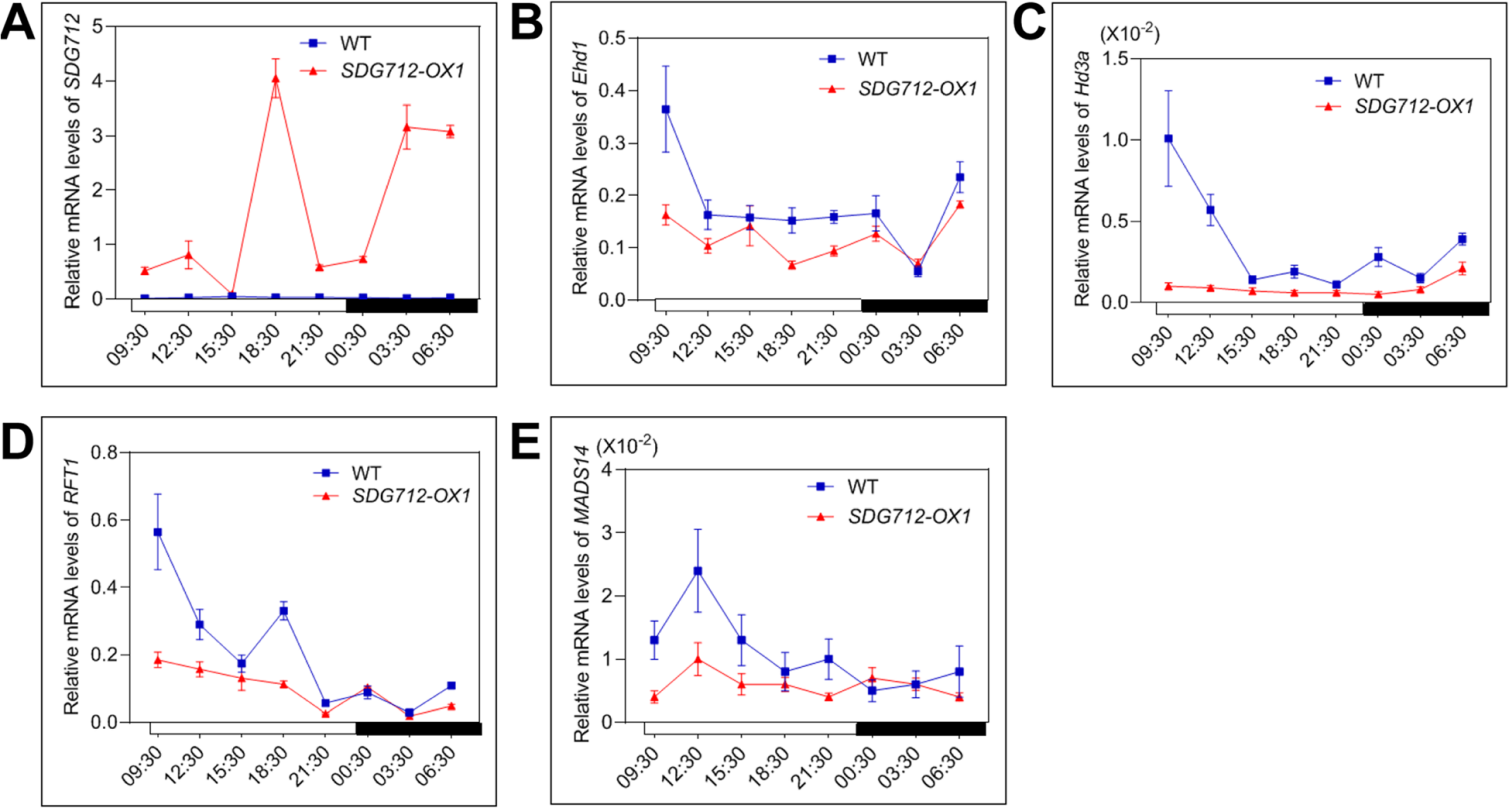
Gene expression under long-day (LD) condition. Plants were grown in artificial climate cabinets under LD condition with 15 h light/9 h dark at 28 °C. Penultimate leaves were collected for RNA extraction every 3 h within 24 h from 50-day-old plants. *OsActin1* gene was used as internal control. WT, Zhonghua11. **A**, Transcript levels of *SDG712* in WT and *SDG712-OX1* plants. **B**-**E**, The expression of *Ehd1*, *Hd3a*, *RFT1* and *MADS14* in WT and *SDG712-OX1* plants. Open boxes denote light-period and filled boxes denote dark-period

Given the change of rice flowering time in *SDG712* overexpression transgenic lines and *sdg712* mutant, we speculated that *SDG712* may affect the expression of genes involved in photoperiodic flowering pathway. To reveal the molecular mechanism of SDG712, we investigated the regulatory relationship between *SDG712* and several key genes of photoperiodic flowering pathway. Firstly, expression of *SDG712* was significantly reduced in *hd1–3* mutant under both SD and LD conditions, suggesting that *Hd1* acts as the upstream regulator to positively regulate the expression of *SDG712* (Fig. 1A, B). Then, we examined the expressions of several key flowering regulatory genes in *SDG712*-*OX* lines and the wild type Zhonghua11. The results showed that the expressions of *Ehd1*, *Hd3a*, *RFT1* and *MADS14* were significantly down-regulated in *SDG712*-*OX* lines under both short-day and long-day conditions (Fig. 5 and Fig. 6), while the expressions of other flowering regulatory genes, including *OsMADS50*, *Ehd2*, *Ehd3*, *Ehd4*, *DTH7*, *DTH8*, *Ghd7* and *Hd1*, were not significantly changed between *SDG712*-*OX* lines and the wild type (Supplemental Fig. 3 and Supplemental Fig. 4). These results indicated that *SDG712* gene functions downstream of *Hd1*, while upstream of key regulator *Ehd1* and the florigen genes.

### SDG712 Protein Is Localized in the Nucleus

**Fig. 7:**
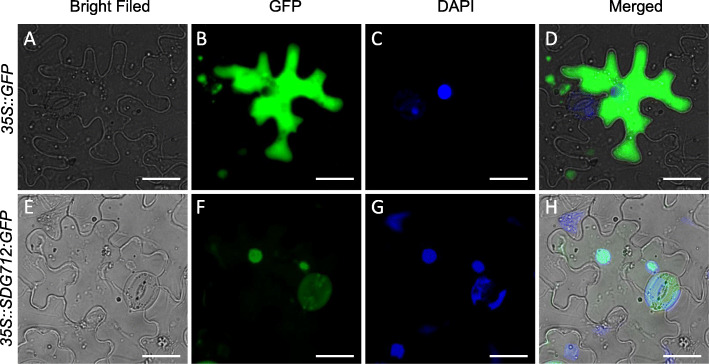
Subcellular localization assay of SDG712 protein. **A**–**D**, The transient expression of pCAMBIA35S::GFP empty vector in tobacco epidermal cells. **E**–**H**, The transient expression of pCAMBIA35S::SDG712:GFP fusion vector in tobacco epidermal cells. Bars = 25 μm

Since *SDG712* encodes a putative H3K9-specific HMTase, which may functions in histone methylation, we speculated that the SDG712 protein should be localized in the nucleus. To test this, we carried out subcellular localization assay of SDG712 protein. The recombinant vector expressing *CaMV35S::SDG712:GFP* was transiently expressed in tobacco, with the empty vector expressing *CaMV35S::GFP* served as control. The results showed that GFP signal was exclusively observed in the nucleus in the epidermal cell of tobacco with recombinant vector, while GFP signal was observed ubiquitously in the cell in the control with empty vector (Fig. 7), suggesting that SDG712 protein is exclusively localized in the nucleus and thus may play roles in the nucleus.

### The H3K9me2 Levels of *Hd3a* and *RFT1* Loci Were Increased in *SDG712* Overexpression Transgenic Plants

**Fig. 8:**
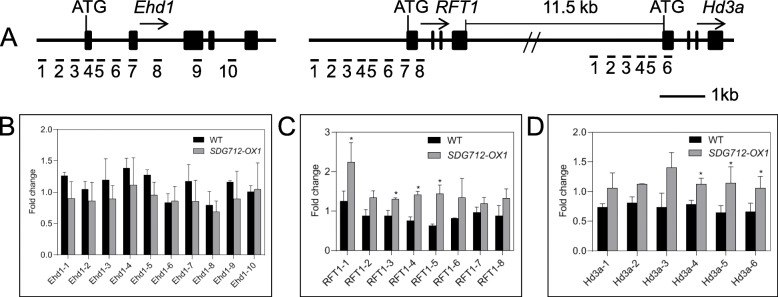
Chromatin immunoprecipitation qPCR (ChIP-qPCR) assays of *Ehd1*, *RFT1* and *Hd3a* loci. Leaves of 50-day-old plants grown in fields were collected 3 h after dawn. ChIP assays were performed using antibodies against histone H3K9 dimethylation (H3K9me2), and qPCR (Quantitative PCR) was used to analyze the levels of methylation of *Ehd1*, *RFT1* and *Hd3a*. **A**, the structure of *Ehd1*, *RFT1* and *Hd3a*. Filled boxes denote exons and lines denote introns or 5′ upstream regions of corresponding genes. Arrows denote the transcription direction of genes. The numbers 1–10 denote the position of primers. **B**-**D**, qPCR results of *Ehd1*, *RFT1* and *Hd3a* loci, respectively. *OsActin1* was used as an internal standard for normalization. WT, Zhonghua11. Three biological replicates were performed. Asterisks indicate statistically significant differences of t-test (*, *P* < 0.05)

Given that *SDG712* encodes putative H3K9-specific histone methyltransferase and negatively regulates the expression of the downstream *Ehd1* and the florigen genes *Hd3a* and *RFT1*, we further performed ChIP assay to examine the methylation level of *Ehd1*, *Hd3a* and *RFT1* loci. The previous studies have shown that H3K9-specific methyltransferase mainly exhibits H3K9me2 activity (Ding et al., 2007), therefore, we examined the H3K9me2 levels at these loci. 10 primer sets in *Ehd1*, 8 primer sets in *RFT1* and 6 primer sets in *Hd3a* were designed to perform ChIP qRT-PCR to analyze the change of histone methylation level (Fig. 8A). The result demonstrated that H3K9me2 levels at *Hd3a* and *RFT1* loci were significantly increased in the *SDG712-OX1* plants compared with wild type plants (Fig. 8C-D), indicating that overexpression of *SDG712* increased H3K9me2 levels at *Hd3a* and *RFT1* loci. No obvious changes of H3K9me2 level at *Ehd1* locus were detected between *SDG712-OX1* plants and wild type plants (Fig. 8B), even though *Ehd1* expression was significantly down-regulated in *SDG712-OX1* plants compared with wild type plants (Fig. 5C and Fig. 6C). Taken together, these results suggested that SDG712 may mediate the H3K9 di-methylation at *RFT1* and *Hd3a* loci, but not at *Ehd1* locus.

## Discussion

**Fig. 9:**
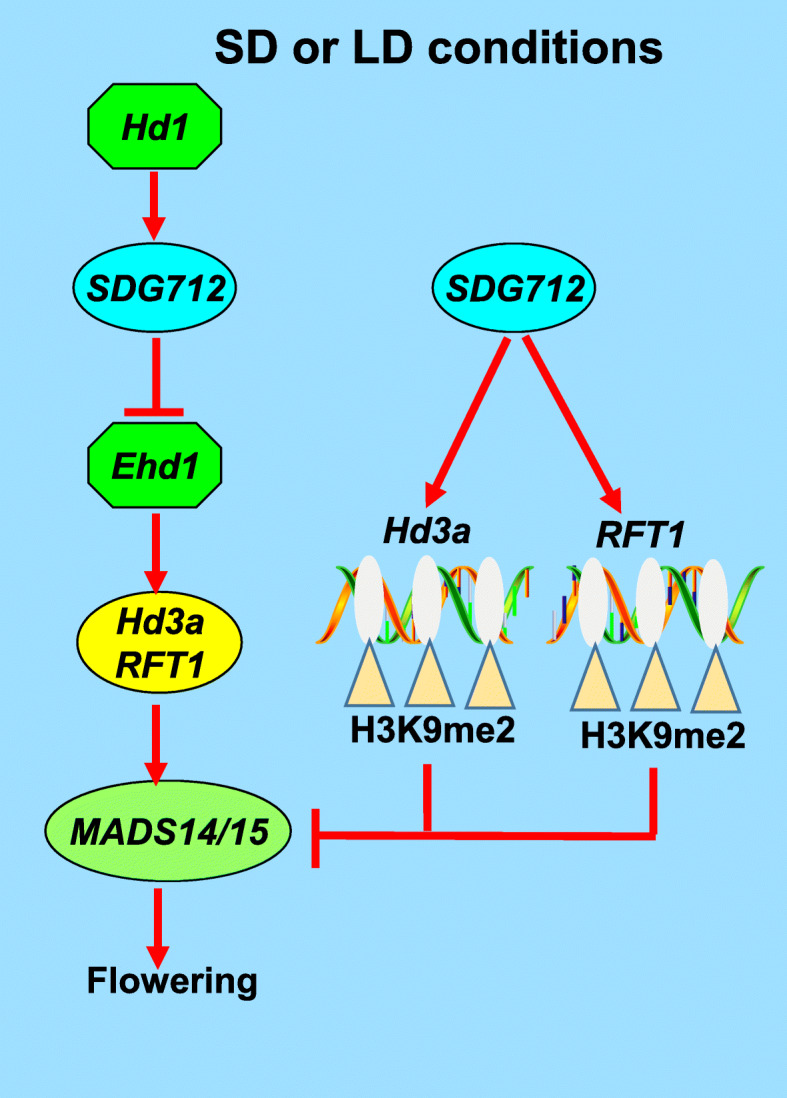
Proposed working model of *SDG712* in rice flowering. *SDG712* negatively regulates rice flowering by two possible pathways. In one pathway, *SDG712* represses the expression of *Ehd1*, thus represses the expression of downstream florigen genes *Hd3a* and *RFT1* to delay rice flowering. In the other pathway, *SDG712* represses the expression of *Hd3a* and *RFT1* genes directly by mediating the H3K9 di-methylation (H3K9me2) at these two loci to delay rice flowering

*SDGs* play important roles in various cellular activities, and regulate plant growth and development. In this study, we identified a histone methyltransferase encoding gene *SDG712* and found that it negatively regulates rice flowering. Loss of function of *SDG712* promotes rice flowering, while overexpression of *SDG712* inhibits rice flowering. Gene expression analysis revealed that the expression of *Ehd1* and the florigen genes *Hd3a* and *RFT1* were down-regulated in *SDG712-OX* plants, resulting in the down-regulation of floral identity gene *MADS14*. Based on these results, we conclude that *SDG712* is involved in the classical photoperiodic flowering regulatory pathway *Ehd1-Hd3a/RFT1-MADS14/15* to regulate rice flowering. In addition, our results showed that *Hd1* functions upstream of *SDG712* and positively regulates expression of *SDG712* under SD and LD conditions. Moreover, *SDG712-OX* lines and *sdg712* mutant has delayed and earlier heading-date, respectively. These results are consistent with the expression patterns of *Hd1* and *SDG712*, which display diurnal expression under SD and LD condition. According to the previous studies, Hd3a is the major florigen under SD condition and RFT1 is the major florigen under LD condition (Komiya et al., 2008), therefore, we propose that the delay of rice flowering under SD condition is mainly controlled by the *Hd1*-*SDG712*-*Ehd1*-*Hd3a*-*MADS14/15* pathway, and under LD condition, it is mainly controlled by *Hd1*-*SDG712*-*Ehd1*-*RFT1*-*MADS14/15* pathway (Fig. 9).

Our ChIP-qPCR assay showed that H3K9me2 levels at *Hd3a* and *RFT1* loci were increased, indicating that *SDG712* inhibits rice flowering through increasing H3K9me2 deposition at *Hd3a* and *RFT1*. Interestingly, H3K9me2 level at *Ehd1* locus was not significantly changed, even though the expression of *Ehd1* was down-regulated in *SDG712*-*OX* plants, suggesting that the reduced expression of *Ehd1* may be caused by other mechanism rather than chromatin modification. This result is similar to other *SDG* members in previous studies. For example, *SDG724* encodes a H3K36-specific HMTase, the transcript level of *Ehd1* was reduced in *sdg724* mutant, but the H3K36me2/3 levels of *Ehd1* were not significantly changed (Sun et al., 2012). *SDG701* encodes a H3K4-specific HMTase, the transcript level of *Ehd1* was reduced in *sdg701–1* mutant, but the H3K4me3 levels of *Ehd1* were not significantly changed (Liu et al., 2017). We propose that *SDG712* can also regulate rice flowering by directly increasing H3K9me2 levels at *Hd3a* and *RFT1* besides the classical photoperiodic flowering regulatory pathway (Fig. 9).

In previous studies, several *SDGs* not only regulated flowering time but also changed the leaf emergence rate or other traits in *Arabidopsis* (Saleh et al., 2008; Tamada et al., 2009). In this study, except heading date, other agronomic traits were not significant difference between wild type and *sdg712* mutant and *SDG712-OX* lines, indicating that *SDG712* gene mainly affecting rice flowering time.

SDGs play important roles in epigenetic regulation of rice flowering. Several different types of SDGs were reported to be involved in this course. H3K4-specific methyltransferase (SDG701 and SDG723) and H3K36-specific methyltransferases (SDG708, SDG724 and SDG725) mainly promote rice flowering, while H3K27-specific methyltransferases (*SDG711*) delays rice flowering (Choi et al., 2014, Liu et al., 2017; Sun et al., 2012; Sui et al., 2013; Liu et al., 2016; Liu et al., 2014). Here, we reported that SDG712, an H3K9-specific methyltransferase, can also delay rice flowering. Based on these studies, SDGs display a complex pattern of epigenetic regulation in rice flowering. In our study, the effects of epigenetic regulation of *SDG712* on rice flowering are relatively weak, since *sdg712* mutants was preceded only 2–3 days under NSD and NLD conditions. It is possible that there may be other unknown H3K9-specific SDG homologs that redundantly regulate rice flowering. There are 15 members of putative H3K9-specific SDGs in rice (Ng et al., 2007); however, the function of the rest 13 members remains unclear. On the other hand, different types of SDGs (H3K4, H3K9, H3K27, H3K36-specific HMTases) may share the same regulatory pathways in flowering to fine-tune the flowering time and to finally fulfill the dynamic balance each other. In addition, beyond SDGs, other genes involved in the process of chromatin modification could also affect the expression of flowering regulatory genes. OsVIL2 is a plant homeodomain (PHD) containing chromatin remodeling factor, it functions together with PRC2 to mediate the H3K27 tri-methylation at *OsLFL1* gene (a repressor of *Ehd1*), thus repressing the *OsLFL1* expression, resulted in promoted rice flowering (Yang et al., 2013). OsVIL3, a homolog of OsVIL2, binds to the promoter region of *OsLF* (a repressor of *Hd1*) and represses the *OsLF* expression via H3K27 tri-methylation, promoting rice flowering under SD condition (Wang et al., 2013). These studies support the notion that in rice flowering is epigenetically regulated and involves complex gene regulatory networks.

## Conclusion

In this study, we identified *SDG712* as a negative regulator of rice heading-date. Mutation of *SDG712* promoted rice flowering, overexpression of *SDG712* delayed rice flowering. *SDG712* acts downstream of *Hd1*, while upstream of florigen gene *Hd3a* and *RFT1*. The di-methylation of H3K9 at *Hd3a* and *RFT1* loci were increased in *SDG712* overexpression plants, demonstrating that SDG712 mediated the H3K9 methylation of the florigen genes. The findings in this study provide an understanding of SDG member’s function.

## Materials and Methods

### Plant Materials

The wild type Nipponbare and *sdg712* mutants; as well as the wild type Zhonghua11 and *SDG712* overexpression transgenic lines were grown in the fields in Tianjin, China (117°E, 39°N; natural long-day condition, NLD) and Lingshui, Hainan province, China (110°E, 18°N; natural short-day condition, NSD) for phenotypic investigation. Plants for diurnal gene expression analysis were grown in artificial climate cabinets with day length 9 h light/15 h dark for short-day condition and 15 h light/9 h dark for long-day condition at 28 °C.

### Affymetrix GeneChip Analysis

Affymetrix Rice Genome Array was used in gene expression analysis. Biotinylated RNAs were prepared according to the standard Affymetrix protocol (Affymetrix, Inc. USA). Affymetrix GenChip hybridization, washing, staining, and scanning were carried out according to manufacturer’s instructions. GeneChips were scanned using Affymetrix Scanner 3000 with default settings. The microarray data were analyzed using Molecule Annotation System (MAS 5.0) algorithm. Microarray data has been deposited in NCBI GEO database under the accession number of GSE166053.

### Characterization of the *sdg712* Mutant

To obtain *SDG712* knockout mutants, we searched the Rice *Tos17* Insertion Mutant Database (https://tos.nias.affrc.go.jp/) and screened the line *NG0566*, in which the *Tos17* fragment was inserted into the fourth exon of *SDG712* gene. We acquired the *NG0566* line and characterized it by tri-primer PCR using *Tos17* specific primer pTail3 and *SDG712* gene specific primers pNG0566-F and pNG0566-R (Supplemental Table 2).

### The Obtainment of Transgenic Plants Overexpressing *SDG712*

For overexpression of *SDG712*, coding sequence was amplified from the total cDNA of Zhonghua11 with primers pSDG712-OX-F and pSDG712-OX-R (Supplemental Table 2), and then cloned into binary vector pCAMBIA2300 with double *CaMV35S* promoter. The recombinant vector was transformed into *Agrobacterium* strain *EHA105* via electroporation transformation method. Subsequently, the transgenic plants were obtained using the *Agrobacterium*-mediated transformation method described by Hiei (Hiei et al., 1994).

### Gene Expression Analysis

Sample collections of gene expression analysis are as follows: (1) For spatial and temporal expression analyses, the wild type Zhonghua11 plants were grown in the paddy field under natural long-day condition. Various tissues were collected at 10 a.m. at different developmental stages: SAMs were collected from 8-day-old plants; leaves, sheaths and roots were collected from 50-day-old plants; panicles were collected from reproductive stage plants. (2) For expression analysis at different growth stages, Zhonghua11 plants were grown in the paddy field under natural long-day condition, penultimate leaves were collected in the morning at 10 a.m. every 10 days from the beginning of 40-day-old Zhonghua11 plants. (3) For diurnal expression analyses, rice plants were grown in artificial climate cabinets under SD condition with 9 h light/15 h dark at 28 °C and under LD condition with 15 h light/9 h dark at 28 °C. Penultimate leaves of 50-day-old plants were collected for RNA extraction every 3 h within 24 h cycle. Three biological replicates were performed.

For all gene expression analysis, total RNAs were extracted using Trizol solution (Invitrogen, USA). cDNAs were synthesized from 1 μg of total RNA. One micro liter of cDNA was used for real-time PCR analysis using SYBR Green PCR master mix (Vazyme biotech, China) and the gene specific primer pairs (Supplemental Table 2). Real-time PCR was performed in a LightCycler480 system (Roche, USA). *OsActin1* gene was used as an internal control. Data were analyzed by the LightCycler480 Software system according to the instruction manual. Relative expression levels were calculated following 2^-ΔΔCt^ method described previously (Livak and Schmittgen, 2001).

### Subcellular Localization

The cDNA sequence of *SDG712* without stop codon was cloned and ligated into the empty vector p35S::GFP to produce the recombinant plasmid p35S::SDG712:GFP. Then, p35S::SDG712:GFP was introduced into *Agrobacterium* strain and infiltrated into tobacco leaves. 72 h after infiltration, the GFP signals in epidermal cell layers were observed under fluorescence microscope (Leica, DM5000B, Germany), and images were captured.

### Chromatin Immunoprecipitation Experiments

Chromatin immunoprecipitation (ChIP) experiments were performed using 50-day-old rice plants grown in paddy field under natural LD condition. The experiment was performed according to the method described previously (Weng et al., 2018). Antibodies against H3K9me2 was purchased from Abcam (Cat no. ab1220). Quantitative real-time PCR was carried out to determine the enrichment of DNA immunoprecipitated in the ChIP experiments, using gene-specific primers of *Ehd1*, *Hd3a* and *RFT1* (Supplemental Table 2; Liu et al., 2017). *OsActin1* was used as internal control.

## Supplementary Information

**Additional file 1: Supplemental Fig. 1.** Microarray gene expression analysis of *hd1–3*. (A) Global gene expression changes in the mutant *hd1–3* compared with the wild-type. Each row represents a rice gene. Red bracket indicate large amounts of genes have slight expression changes. The log_2_ (*hd1–3*/WT) value is presented in color scale. LD, long-day; SD, short-day. (B) Gene ontology analysis of the genes having 2-fold change under both LD and SD conditions. (C) 44 genes show > 5-fold changes under both LD and SD conditions. The red asterisk indicates *SDG712* (*LOC_Os02g40770*)*.* **Supplemental Fig. 2.** Agronomic traits of *sdg712* and the overexpression lines under natural long-day condition. ZH11, Zhonghua11; NIP, Nipponbare. *N* ≥ 30. **Supplemental Fig. 3.** Gene expression analysis under short-day (SD) condition. Plants were grown in artificial climate cabinets under SD condition with 9 h light/15 h dark at 28 °C. Penultimate leaves were collected for RNA extraction every 3 h within 24 h from 50-day-old plants. Os*Actin1* gene was used as internal control. WT, Zhonghua11. Open boxes denote light-period and filled boxes denote dark-period. **Supplemental Fig. 4.** Gene expression analysis under short-day (LD) condition. Plants were grown in artificial climate cabinets under SD condition with 9 h light/15 h dark at 28 °C. Penultimate leaves were collected for RNA extraction every 3 h within 24 h from 50-day-old plants. Os*Actin1* gene was used as internal control. WT, Zhonghua11. Open boxes denote light-period and filled boxes denote dark-period.

**Additional file 2: Supplemental Table 1.** Global gene expressions in wild-type rice and hd1–3 mutant.

**Additional file 3: Supplemental Table 2.** Primers used for vector construction and gene expression analysis.

## Data Availability

The datasets supporting the conclusions of this article are included within the article and its additional files.

## Abbreviations

ChIP: Chromatin immunoprecipitation
DAG: Days after germination
FAC: Florigen activation complex
HMTase: Histone methyltransferases
LD: Long-day condition
NLD: Natural long-day condition
NSD: Natural short-day condition
SAM: Shoot apical meristem
SD: Short-day condition
SDG: SET Domain group
