# Supplementary figures and images for: SDG712, a Putative H3K9-Specific Methyltransferase Encoding Gene, Delays Flowering through Repressing the Expression of Florigen Genes in Rice
Source: Rice (N Y). 2021 Aug 6;14:73. doi: 10.1186/s12284-021-00513-9 (PMC8346621; doi:10.1186/s12284-021-00513-9)

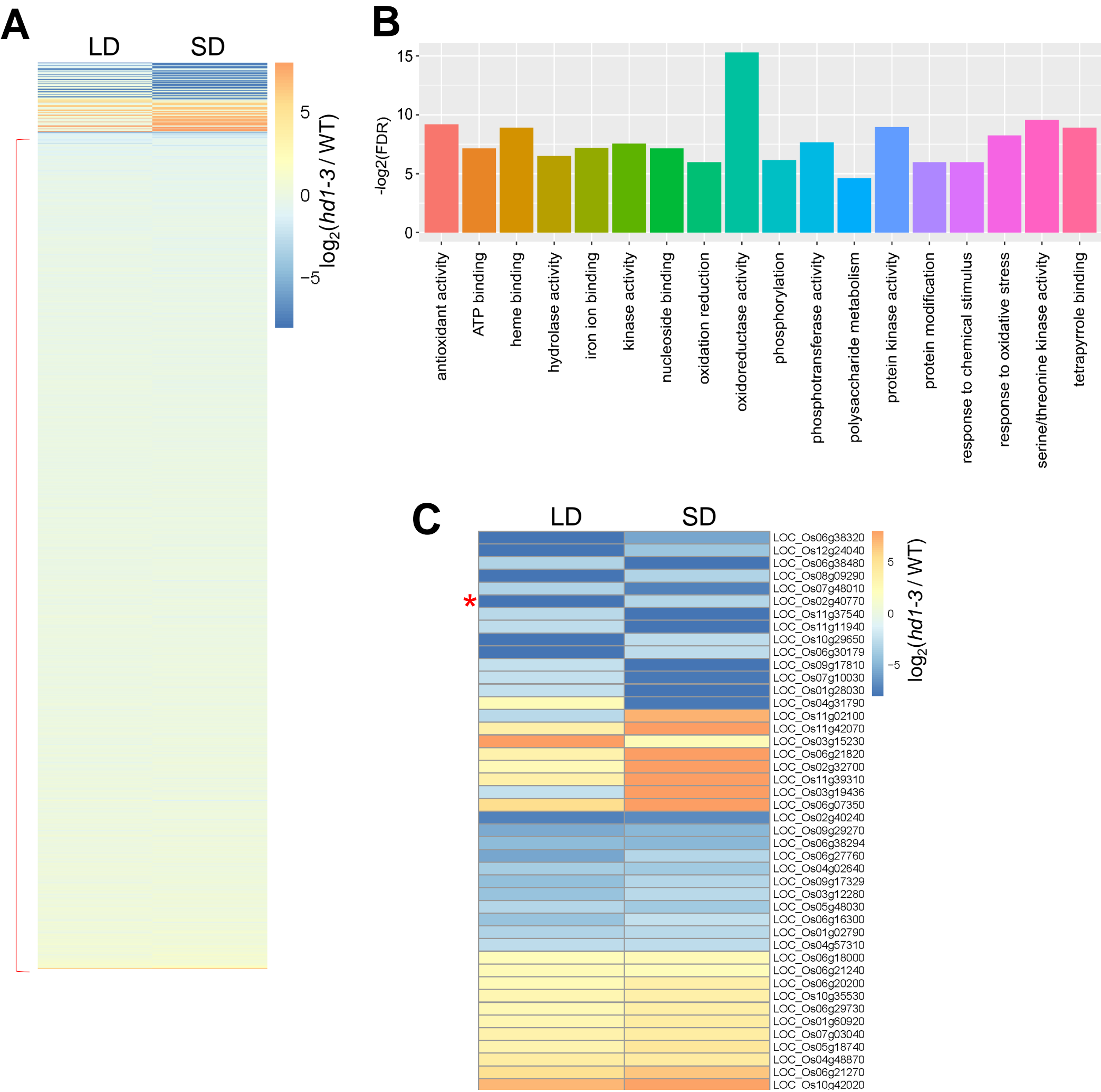

Supplemental Figure 1

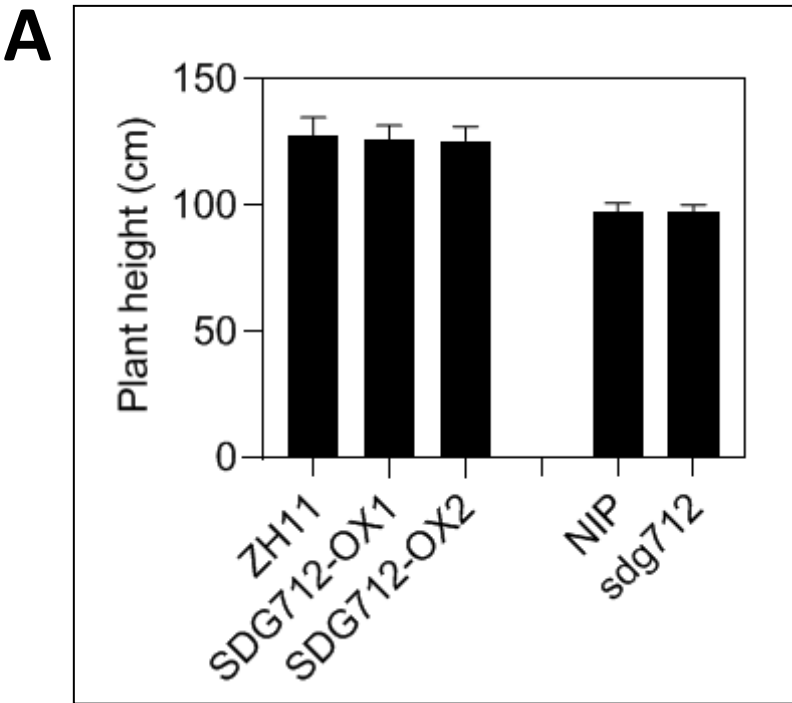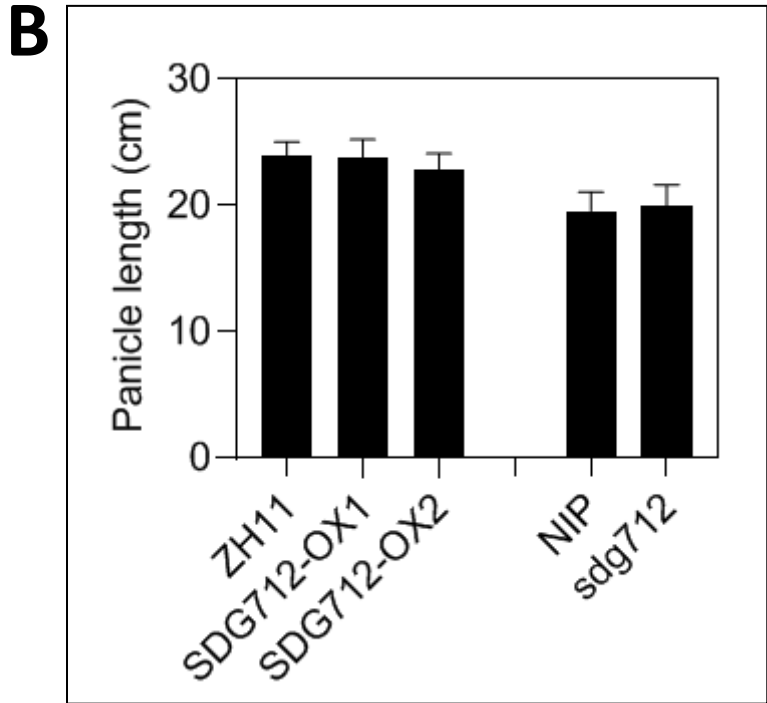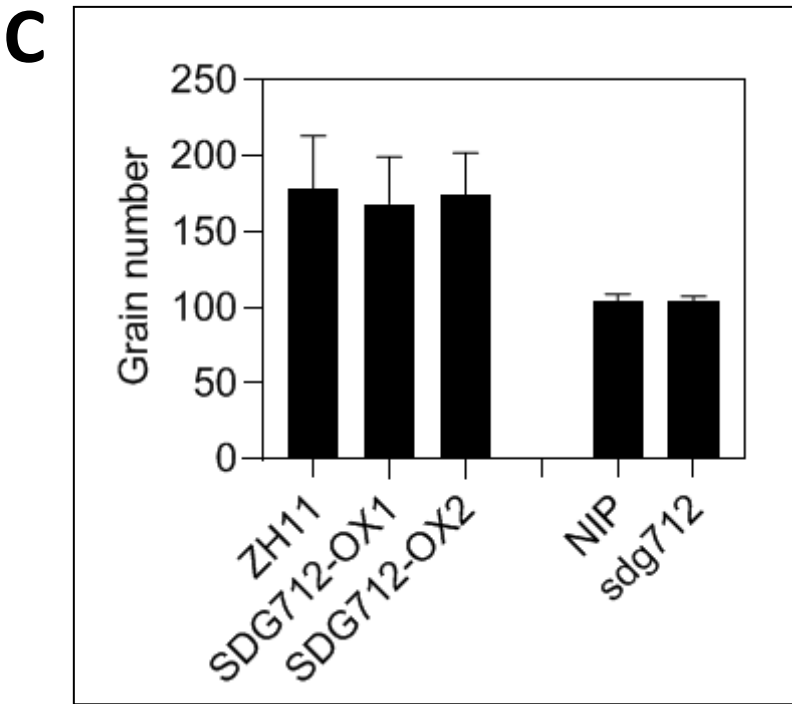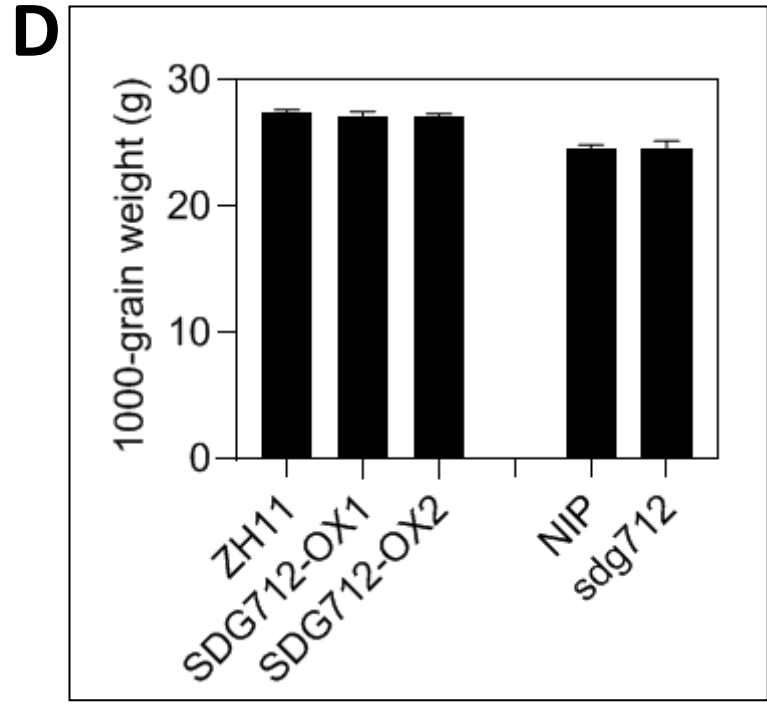

Supplemental Figure 2

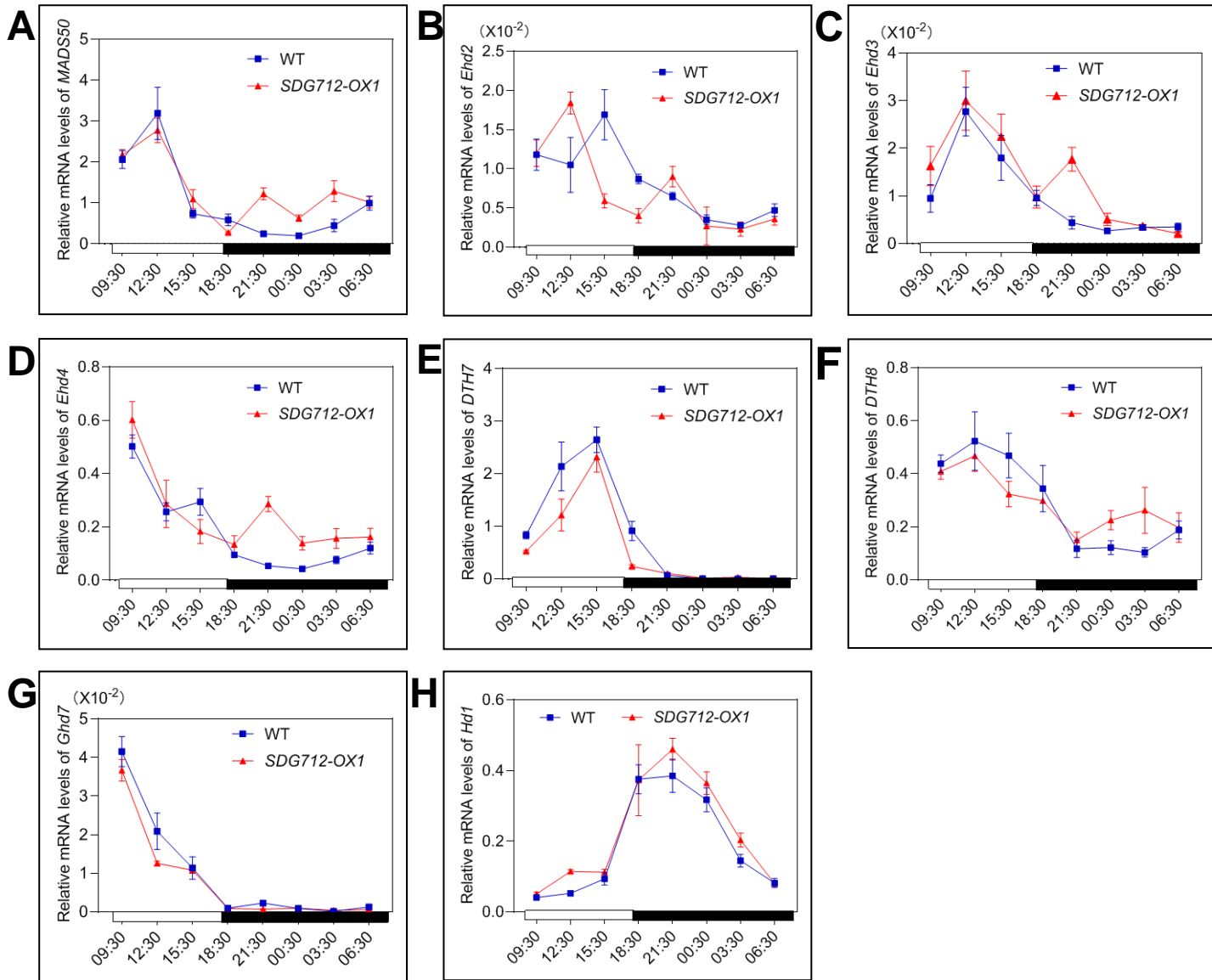

Supplemental Figure 3

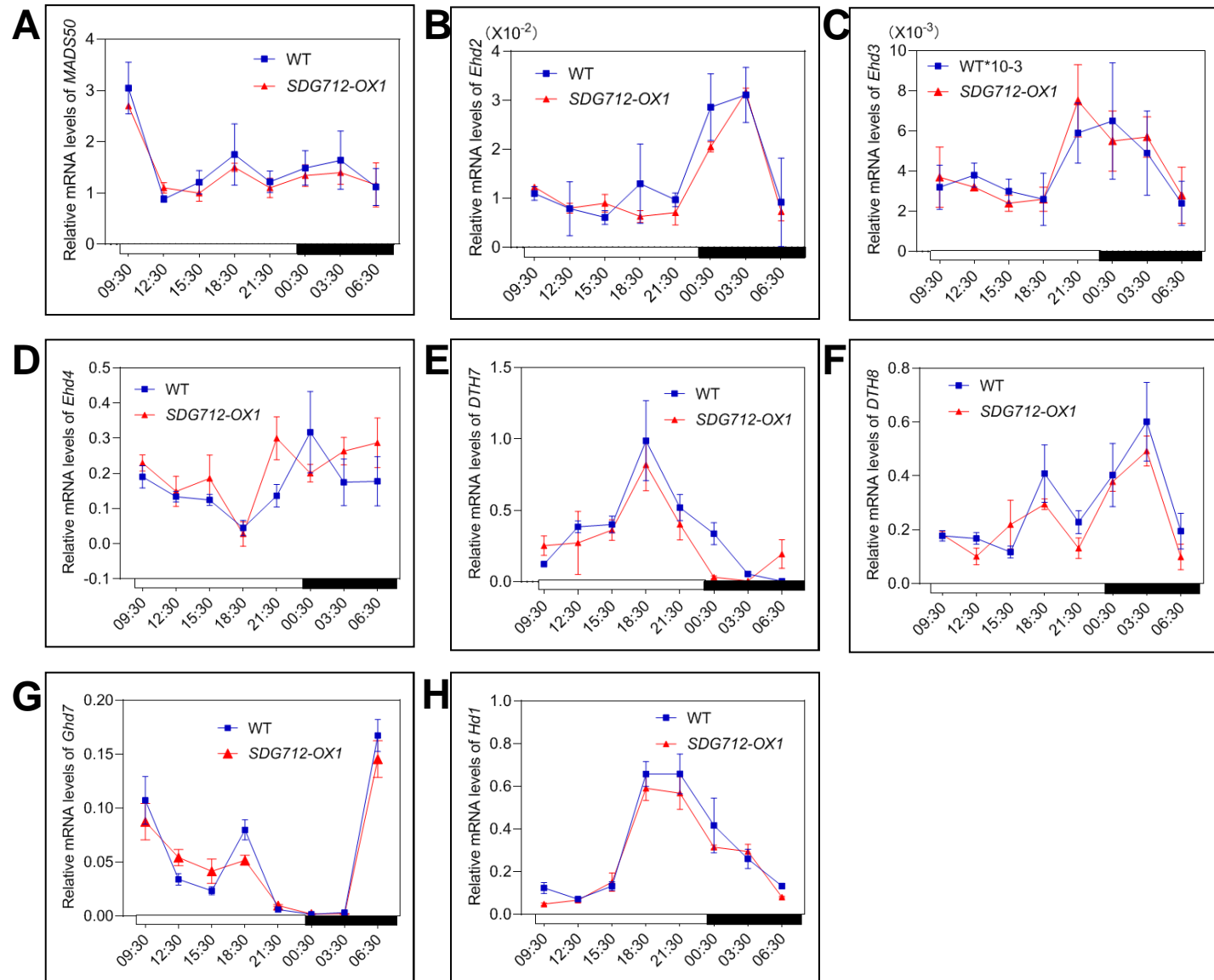

Supplemental Figure 4

Supplement: Supplementary file 1 — Additional file 1: Supplemental Fig. 1. Microarray gene expression analysis of hd1–3. (A) Global gene expression changes in the mutant hd1–3 compared with the wild-type. Each row represents a rice gene. Red bracket indicate large amounts of genes have slight expression changes. The log2 (hd1–3/WT) value is presented in color scale. LD, long-day; SD, short-day. (B) Gene ontology analysis of the genes having 2-fold change under both LD and SD conditions. (C) 44 genes show > 5-fold changes under both LD and SD conditions. The red asterisk indicates SDG712 (LOC_Os02g40770). Supplemental Fig. 2. Agronomic traits of sdg712 and the overexpression lines under natural long-day condition. ZH11, Zhonghua11; NIP, Nipponbare. N ≥ 30. Supplemental Fig. 3. Gene expression analysis under short-day (SD) condition. Plants were grown in artificial climate cabinets under SD condition with 9 h light/15 h dark at 28 °C. Penultimate leaves were collected for RNA extraction every 3 h within 24 h from 50-day-old plants. OsActin1 gene was used as internal control. WT, Zhonghua11. Open boxes denote light-period and filled boxes denote dark-period. Supplemental Fig. 4. Gene expression analysis under short-day (LD) condition. Plants were grown in artificial climate cabinets under SD condition with 9 h light/15 h dark at 28 °C. Penultimate leaves were collected for RNA extraction every 3 h within 24 h from 50-day-old plants. OsActin1 gene was used as internal control. WT, Zhonghua11. Open boxes denote light-period and filled boxes denote dark-period. [file 12284_2021_513_MOESM1_ESM.pdf]
